# Testing the diagnostic expansion hypothesis with a population-based survey of attitudes to depression in Australia

**DOI:** 10.1136/bmjph-2025-003040

**Published:** 2025-09-09

**Authors:** Nicola Reavley, Anthony Jorm, Stephen Carbone, Ellie Tsiamis, Amy Joanna Morgan

**Affiliations:** 1 Centre for Mental Health and Community Wellbeing, Melbourne School of Population and Global Health, The University of Melbourne, Melbourne, Victoria, Australia; 2 Prevention United, Melbourne, Victoria, Australia

**Keywords:** Mental health literacy, depression, diagnostic expansion, population survey

## Abstract

**Introduction:**

There is growing concern about the increasing use of psychiatric terminology to describe behaviours and experiences that do not meet criteria for diagnosis of a mental illness. We aimed to conduct a nationally representative online cross-sectional survey exploring diagnostic labelling of vignettes describing a person with different levels of depression symptoms or risk and whether this was associated with mental health-related intended actions and psychological distress.

**Methods:**

Australian respondents (n=6142) were randomly assigned to read one of five vignettes describing a person in the following situations: (1) currently well (family history of depression), (2) currently well (own history of depression), (3) subthreshold depressive symptoms, (4) major depressive disorder (MDD) and (5) MDD with suicidal thoughts. They were asked what, if anything, was wrong with this person. Further questions covered intentions to seek professional help or take self-help actions; psychological distress and personal experience of depression.

**Results:**

Labelling non-clinical or subthreshold vignettes with diagnostic labels was relatively common, with a depression label applied by 19.8% [99% CI 16.6, 23.6], 31.3% [99% CI 27.4, 35.6], 47.7% [99% CI 43.4, 52.0], 68.6 [99% CI 64.5, 72.5] and 77.2 [99% CI 73.1, 80.7] of respondents to vignettes 1 to 5 respectively. Younger people were more likely to give a depression label. Across all vignettes, labelling was associated with a greater likelihood of intentions to speak to a health professional or take medication but not with psychological distress or reductions in effective self-help.

**Conclusions:**

Findings suggest that public messages should have a more nuanced approach, making it clear that, for some mental health difficulties, non-medical solutions may be more appropriate, while also taking care not to increase the proportion of people with more severe problems who meet diagnostic criteria but do not seek help.

**WHAT IS ALREADY KNOWN ON THIS TOPIC**

- While there is growing concern about the increasing use and consequences of psychiatric terminology to describe behaviours and experiences that do not meet criteria for diagnosis of a mental illness, there are no population surveys using large, nationally representative samples that have explored this and its potential consequences.

**WHAT THIS STUDY ADDS**

- The study adds robust support for the hypothesis that expanded concepts of depression are common, particularly in younger people, and that these are associated with intentions to speak to a health professional or take medication for non-clinical issues.

**HOW THIS STUDY MIGHT AFFECT RESEARCH, PRACTICE OR POLICY**

- Our study adds to research highlighting the need for strengthening public messages and narratives about non-clinical solutions to distress, including evidence-based self-care strategies, or low-intensity psychosocial supports.

## Introduction

 Despite the high global prevalence of mental health problems, a substantial proportion of individuals experiencing psychological distress do not seek professional help—a phenomenon commonly referred to as the help-seeking or treatment gap.^1^ Estimates suggest that over half of those affected by common mental health problems such as depression and anxiety do not access formal support.^2^ Multiple interrelated factors contribute to this underutilisation, including structural barriers such as cost, accessibility and service availability, as well as individual beliefs and attitudes.^3^ Sociodemographic disparities further compound the issue, with lower rates of help-seeking observed among men, young people and individuals from culturally and linguistically diverse backgrounds.

In the latter half of the 20th century, it was recognised that closing the mental health treatment gap would require action in both clinical and population settings.^4^ Prior to this, efforts had largely focused on training health practitioners to better identify and manage mental health problems. In 1997, Jorm and colleagues coined the term ‘mental health literacy’, defined as “knowledge and beliefs about mental disorders which aid their recognition, management or prevention”.^5^ Surveys in multiple countries showed that the general public had poorer mental health literacy than health professionals.^6^ As a result of this, public education, which incorporates efforts to raise awareness about mental illness, provision of information on signs, symptoms and treatments and encouragement of help-seeking, is now a central element of mental health policy and practice.^7^ Educational interventions are commonly integrated with anti-stigma reduction efforts. These may overlap in goals, strategy and content, particularly in relation to stigma as a barrier to help-seeking, although the latter often have a greater focus on changing attitudes and reducing prejudice towards individuals.^8^

As a result of these efforts, recognition of mental illness and beliefs about appropriate sources of help have moved closer to those of health professionals. Nationally representative surveys conducted in Australia showed that, over a 16-year period, levels of depression recognition and beliefs in the helpfulness of mental health professionals and antidepressants increased.^9 10^ Some aspects of stigmatising attitudes have also reduced, particularly those attributing personal weakness to people with more prevalent mental illnesses, notably depression.^6 11^

The approaches used in mental health education and anti-stigma initiatives have changed over time, with a move away from provision of basic facts about diagnoses and symptoms towards a focus on ‘person-first’ language, mental wellbeing and a shared human experience of mental health.^12^ Campaigns that promote help seeking often aim to increase recognition of symptoms and reduce barriers to treatment by framing a wide range of emotional and psychological experiences as potentially indicative of a diagnosable condition. In parallel, anti-stigma campaigns typically avoid clinical language due to the view that terms such as ‘mental disorders’ or ‘mental illness’ problematise individuals, reinforce negative stereotypes and increase stigma and discrimination, while terms such as ‘mental health issues’ are less likely to do so.^8^

There is growing concern that these approaches may have contributed to an expanded concept of mental illnesses, including depression. Haslam and colleagues^13^ have discussed this in terms of ‘concept creep’ which incorporates the expansion of psychological or harm concepts to new phenomena and to less extreme versions of already known phenomena. Others have referred to psychiatrisation, or the process of defining and treating problems as medical that were previously seen as non-medical, in this case, the pathologising or psychiatric framing of everyday life experiences.^14^ Beeker *et al*^15^ note the parallels with ‘psychologization’ and the development of ‘therapy culture’ which involves growing interest in individual emotions and psychological mechanisms.

This complex phenomenon represents an interaction between individuals, society and the psychiatry and psychology professions and may be ‘top down’ and ‘bottom up’.^15^ Top-down processes are those driven by health professionals, pharmaceutical companies and policymakers, for example, the diagnostic expansion represented by a growth in diagnoses or addition of new disorders in the 5th edition of the Diagnostic and Statistical Manual of Mental Disorders (DSM-5).^16^ Bottom-up processes can be seen as those driven by non-professionals. They may include efforts to make sense of experiences through clinical diagnosis, the need to obtain diagnoses to access support or advocacy efforts on behalf of people with particular diagnoses.

While there is evidence that expanded concepts of mental illness may be associated with lower levels of stigmatising attitudes,^17 18^ there is growing concern that expanded concepts of mental illness might have negative impacts. On an individual level, some have argued that labelling people, particularly young people, with illness labels may change their identity in fundamental ways, leading them to reshape their experiences and emotions, potentially increasing feelings of vulnerability and sensitivity to symptoms.^15 19^ It is hypothesised that this can contribute to disempowerment, dependence on medical intervention, rather than on non-clinical or self-help approaches. It may even increase distress and the prevalence of mental health conditions, a phenomenon termed the prevalence inflation hypothesis.^20 21^ It is arguable that the rise of social media has exacerbated this phenomenon due to the proliferation and popularity of content from users with self-identified mental health illnesses.^22^

At a health system level, negative impacts of expanded concepts of mental illness may include over-diagnosis and inappropriate treatment, including side-effects from unnecessary medications or the use of short-term medical interventions for social problems.^23^ There is also concern about the misallocation of health system resources, with services adapted to people with mild or non-specific symptoms, or resources diverted from those with more severe illnesses.^24^ At a societal level, there is a risk that ‘pathologizing’ everyday distress puts the emphasis on supporting affected individuals using clinical interventions rather than using political solutions to address the social determinants that contribute to people’s distress.^15^

Given the potential downsides of expanded concepts of mental illness, it is striking that there has been relatively little empirical research in the area. Recent cross-sectional studies, including one with 298 participants recruited from the Prolific survey platform, showed that people with broader concepts of disorder were more likely to report lower stigma, more positive help-seeking attitudes and self-reported mental health problems.^18^ Concept breadth was also associated with younger age. Another study of 474 participants from Prolific showed that participants with broader concepts of disorder were more likely to self-diagnose and that self-diagnosis was strongly associated with recent formal help-seeking behaviours.^25^ In an experimental study, Speerforck *et al*^26^ examined a mechanism by which mental illness concepts might expand. They asked 138 students to classify statements about a person according to whether they indicated mental illness. The experiment varied the proportion of statements that clearly indicated mental illness. Students presented with a lower proportion of clear mental illness statements were more likely to classify healthy and ambiguous statements as indicating mental illness, indicating that their concept of mental illness had expanded. This may mean that interventions that use vague descriptions of mental illness contribute to diagnostic expansion.

However, no previous studies have investigated the impact of this phenomenon on self-help actions or individual agency and there are no studies with large, methodologically rigorous, nationally representative samples more appropriate for robust population estimates.^27^ These would also enable further exploration of age cohort effects, which is particularly relevant in the context of increasing prevalence of mental health conditions in younger people, as well as more widespread dissemination of mental health literacy programmes in schools.^28^

The aim of the study was to explore knowledge and attitudes about expanded concepts of depression, including (1) the extent to which participants label non-clinical and subthreshold vignettes with diagnostic labels; (2) any associations between labelling and age cohort, gender and education level and country of birth; (3) whether the labelling of non-clinical and subthreshold vignettes with diagnostic labels was associated with psychological distress, as well as the likelihood of taking self-help actions and seeking professional help. We hypothesised that labelling of non-clinical and subthreshold vignettes with diagnostic labels would be associated with psychological distress, a lower likelihood of taking self-help action and a higher likelihood of seeking professional help.

## Materials and methods

Online surveys were completed by 6142 members of the general Australian community aged 16 and over. The survey was carried out by the survey company The Social Research Centre, using their Life in Australia probability-based panel (i.e., a national survey panel recruited using random sampling from an entire population). Panel members were sent an initial survey invitation via email and SMS (where available), followed by up to five reminders within the fieldwork period. Those aged 16–17 years old were invited via their parents’ emails (with parent permission), with two reminders administered. All panel members were offered a reimbursement of AUD$10 for completing the survey (either in the form of a gift card or a donation to a charity). The average survey length was 18.3 min. Data collection was completed in August and September 2024.

## Survey questionnaire

Initial questions covered sociodemographic information: age, gender, Aboriginal and Torres Strait Islander status, country of birth, highest level of primary/secondary school completed, level of the highest educational qualification, employment status (in work/on leave/unpaid/not in work and full time/part time/casual/self-employed). Respondents were also asked if they had completed a training course in how to help someone experiencing a mental health problem, including professional training (with specified options of psychologist, psychiatrist, nurse, general practitioner, social worker, counsellor and occupational therapist) and other types of training [specifically Mental Health First Aid (MHFA), Applied Suicide Intervention Skills Training (ASIST) or Question, Persuade Refer (QPR) or Other (please specify)].

### Mental health and psychological distress

Psychological distress was measured using the Kessler 10 (K10) Psychological Distress Scale Plus,^29^ which comprises 10 questions asking about the frequency of depression and anxiety symptoms over the past 4 weeks. Scores of 10–15 represented low distress and scores of 16–50 as some distress.^30^ Other questions covered the number of days and half days out of role because of any feelings of psychological distress.

Respondents were also asked whether, over the last 12 months, they had experienced any sort of mental health problem (defined as “a period of weeks or more when you are feeling depressed, anxious, or emotionally stressed, and these problems are interfering with your life. Mental health problems could include, for example, depression, anxiety disorders, eating disorders, schizophrenia, bipolar disorder, or personality disorders”).

### Vignettes describing a person’s mental health status

Respondents were then randomly assigned to read one of five vignettes, with the gender of the person in the vignette matched to respondent gender (see online supplemental appendix A). The vignettes described a person ‘Sam’ in the following situations: (1) currently well (family history of depression), (2) currently well (own history of depression), (3) subthreshold depressive symptoms, (4) major depressive disorder (MDD) and (5) MDD with suicidal thoughts. Vignette 3 was based on the DSM-5 definition of subsyndromal (here termed subthreshold) depression as including two or more symptoms of depression that have been present for at least 2 weeks, as well as impaired social functioning. Vignettes 4 and 5 were written to satisfy the DSM-5 and the International Classification of Diseases 11th Revision (ICD-11) diagnostic criteria for major depression and were used in the 2011 National Survey of Mental Health Literacy and Stigma.^31^ The non-clinical and subthreshold vignettes were adapted for this survey to align with vignettes 4 and 5. After being presented with the vignette, respondents were asked what, if anything, they thought was ‘wrong’ with the person described in the vignette.

### Intended actions and beliefs

The vignettes were followed by a series of questions about the likely actions they would take if they were in Sam’s situation, assessed on a 5-point Likert scale (1=Very unlikely to 5=Very likely). These included formal help-seeking actions adapted from those in the 2020–2022 National Survey of Mental Health and Wellbeing^32^ (e.g., speak to a health professional (e.g., general practitioner (GP), psychologist or psychiatrist); take medication (e.g., antidepressant)). Self-help behaviours (e.g., engage in exercise or physical activity or try to remain involved in purposeful activities for at least a small part of every day) were drawn from Delphi consensus studies on effective self-help strategies for depression and anxiety.^33 34^ Items were summed to generate total scale scores for each of professional help-seeking (4 items) and self-help actions (17 items). Scale ranges were between 4 and 20 and 17 to 85 respectively. Both scales demonstrated acceptable reliability (omega values were 0.76 and 0.93, respectively). These were supplemented by two non-recommended items (wait and see if you improve with time and try to avoid situations that make you feel anxious or uncomfortable).^35^

### Coding of open-ended responses

Responses were coded based on adaptations of pre-coded categories used in previous surveys^31^ and included depression, mental health problems/issues/conditions, anxiety, at risk/developing/early signs of depression or anxiety, physical/ medical illness, suicide, nothing or other. Responses were coded with a ‘yes’ or ‘no’ in each category, so that multiple categories were possible. Responses were coded by one person (ET) with any responses other than the unambiguous (eg, ‘depression’, ‘mental illness’) discussed as a group and consensus obtained.

### Weighting

Weighting procedures involved computation of a base weight and subsequent adjustment based on population benchmarks for specific demographic characteristics. The base weight for each respondent was the product of three weights: (1) enrolment weight, accounting for the initial chances of selection and subsequent post-stratification to key demographic benchmarks; (2) an adjustment for the probability of selection into the sample of the survey; and (3) their response propensity weight, estimated from enrolment information available for both respondents and non-respondents to the survey. The base weight was adjusted to satisfy population benchmarks for the following: age, gender, higher education (Bachelor degree), language other than English spoken at home, state or territory, geographic location (all based on the 2021 Census, supplemented by the latest demographic statistics)^36^ and the number of adults in the household (based on the 2020–2021 National Health Survey).^37^

### Statistical analysis

The data were analysed using per cent frequencies and 99% CIs. Logistic regression analyses were conducted to examine whether having experience of mental health problems or having undertaken mental health training (including mental health professional or other mental health or suicide prevention training such as MHFA) was associated with giving diagnostic labels to the vignettes. Linear regression analyses were conducted to examine whether depression labelling was associated with the likelihood of intended self-help actions and professional help-seeking. Both logistic and linear regression analyses controlled for age, gender, country of birth and level of education. A significance level of p<0.01 was used to reduce Type 1 error resulting from multiple comparisons. All analyses were performed using StataSE 18 (Stata Corp LP, Texas, USA).

## Results

In total, 6142 questionnaires were completed. The completion rate, defined as the proportion of all Life in Australia members invited to participate in this survey, was 63.5% (Life in Australia adult population=66.8%; 16–17-year-olds = 21.1%). There were no missing data, although for some variables, a small proportion of participants ‘Refused’ (no more than 0.5% for any variable). These participants were dropped from these analyses.

Overall, 64.0% [99% CI 62.1, 65.8] of participants were in paid work and 36.0% [99% CI 34.2, 37.9] were not in paid work. Those who had completed health professional training (including mental health professionals, allied health professionals, medical doctors and pharmacists) numbered 10.5% [99% CI 9.4, 11.7]. Additionally, 10.5% [99% CI 9.4, 11.7] had undertaken other mental health training (primarily Mental Health First Aid), and 2.7% [99% CI 2.1, 3.4] had undertaken specific suicide prevention training.

### Prevalence of depression labelling

**Figure 1:**
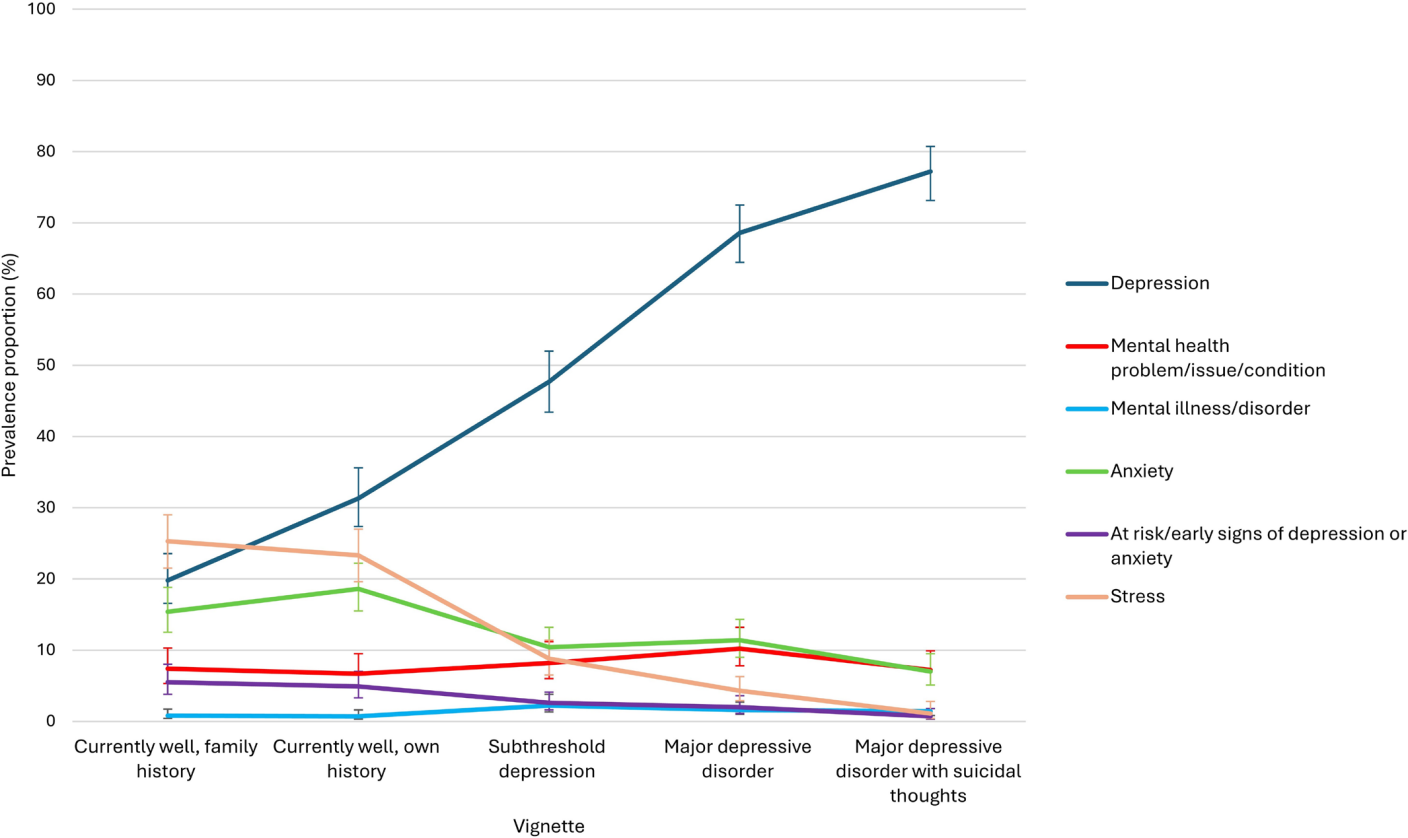
Labels given to the person in the vignettes. Note: error bars represent 99% CIs.

Figure 1 shows the percentage of respondents mentioning the categories to describe the problems shown in the vignettes (only including those nominated by >5% of respondents). Labelling of non-clinical and subthreshold vignettes with a depression label was common, with 19.8% [99% CI 16.6, 23.6] labelling vignette 1, 31.3% [99% CI 27.4, 35.6] labelling vignette 2, 47.7% [99% CI 43.4, 52.0] labelling vignette 3, 68.6 [99% CI 64.5, 72.5] labelling vignette 4 and 77.2 [99% CI 73.1, 80.7] labelling vignette 5. The proportions of people using the depression label increased with the severity of the problem described. Using anxiety and stress labels was more common for vignettes 1 and 2 than the other vignettes.

### Associations with sociodemographic variables

**Figure 2:**
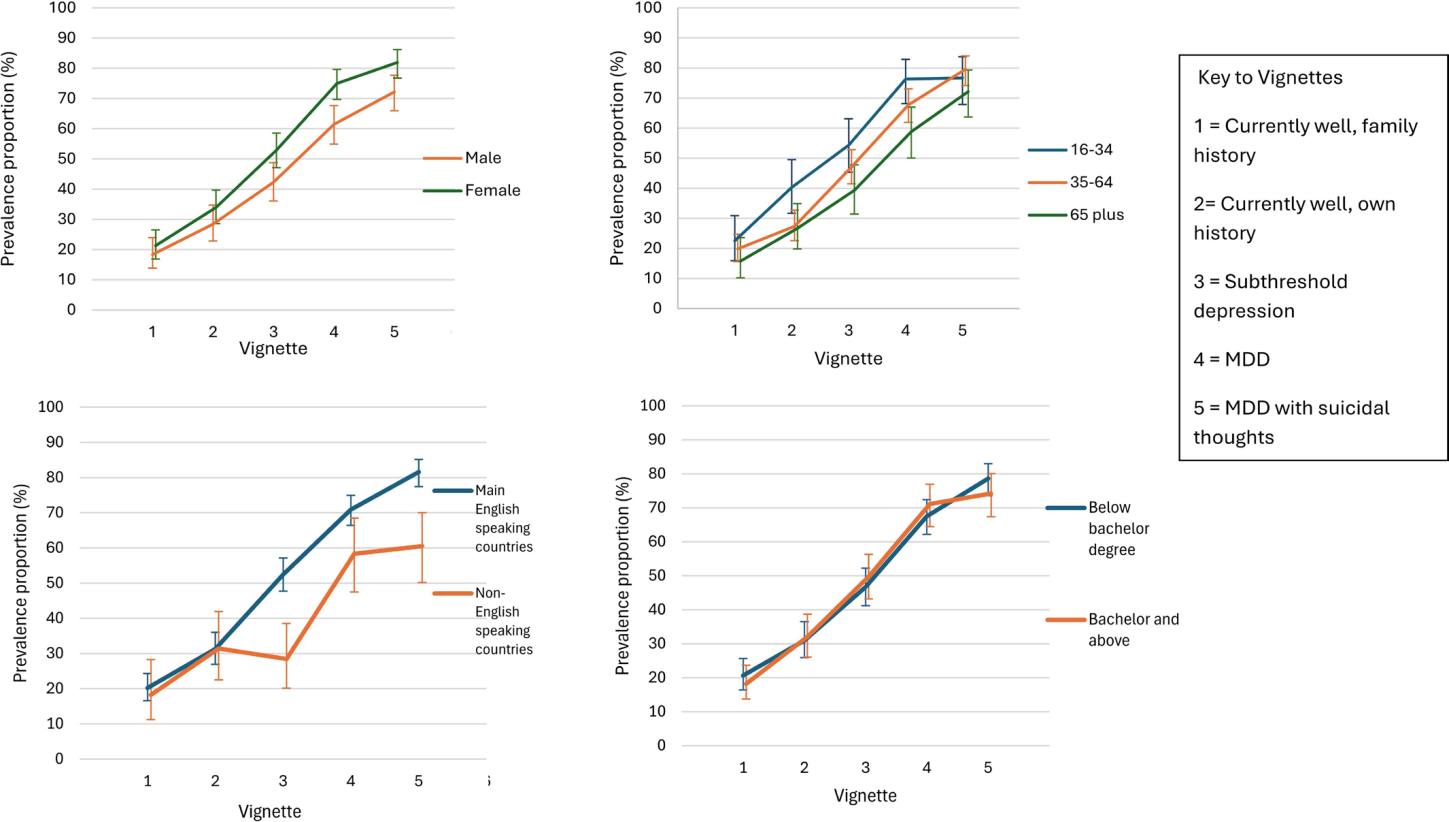
Labels given to the person in the vignette by sociodemographic characteristics of age, gender, country of birth and education level. Note: error bars represent 99% CIs. Respondents preferring a term other than male or female are not shown here due to small sample size and large overlapping CIs.

Females were more likely than males to label vignette 4 with a depression label (see figure 2) and there were no significant differences between age groups. Those born in non-English-speaking countries were less likely to label vignettes 3 and 5 (see figure 2). There were no differences according to level of education (see figure 2). In the multivariable model, females were more likely to label vignettes 4 and 5, those aged 16–34 were more likely than those aged 65 plus to label vignettes 2 and 3, those aged 35–64 were more likely to label vignette 5 and those born in non-English-speaking countries were less likely to label vignettes 3, 4 and 5 (see table 1 and online supplemental table S1).

**Table 1:** Sociodemographic characteristics associated with depression labelling

	Currently well, family history	Currently well, own history	Subsyndromal depression	MDD	MDD with suicidal thoughts
	OR (99% CI)	OR (99% CI)	OR (99% CI)	OR (99% CI)	OR (99% CI)
Gender (reference category: male)					
Female	1.14 (0.73, 1.79)	1.20 (0.81, 1.78)	1.36 (0.95, 1.95)	1.59 (1.06, 2.36)**	1.64 (1.04, 2.59)**
Prefer another term	1.33 (0.08, 23.22)	2.68 (0.39, 18.29)	3.48 (0.46, 26.48)	1.67 (0.33, 8.35)	1.00 (0.00, 0.00)
Age groups (reference category: age 65 plus)					
16–34	1.54 (0.78, 3.02)	1.77 (1.01, 3.10)**	1.85 (1.10, 3.12)**	2.19 (1.26, 3.81)***	1.32 (0.71, 2.45)
35–64	1.33 (0.74, 2.40)	0.99 (0.61, 1.59)	1.40 (0.92, 2.13)	1.49 (0.94, 2.36)	1.71 (1.02, 2.88)**
Country of birth (reference category: Australian born)					
Non-English-speaking countries	0.87 (0.46, 1.63)	1.00 (0.59, 1.70)	0.32 (0.20, 0.53)***	0.52 (0.31, 0.87)***	0.36 (0.21, 0.60)***
Main English-speaking countries	1.07 (0.53, 2.14)	1.27 (0.71, 2.27)	0.81 (0.48, 1.37)	0.92 (0.51, 1.67)	1.27 (0.60, 2.68)
Mental health training (reference category: no training)					
Health professional training	0.95 (0.49, 1.85)	0.97 (0.53, 1.76)	1.50 (0.80, 2.81)	2.01 (0.91, 4.44)	1.41 (0.65, 3.07)
Mental health/suicide prevention training	1.05 (0.53, 2.06)	1.50 (0.86, 2.63)	1.49 (0.87, 2.52)	1.21 (0.65, 2.24)	0.91 (0.42, 2.01)

**p<0.01, ***p<0.001 (in bold type).

MDD, major depressive disorder.

Having health professional training, or any suicide prevention specific or mental health training, was not associated with labelling when controlling for sociodemographic variables of age, gender, country of birth and level of education (see table 1 and online supplemental table S1).

### Associations with intended actions

Mean (SE) scores were as follows: professional help-seeking: M (SE)=12.0 (0.06) and self-help actions: M (SE)=65.8 (0.17).

Labelling depression was associated with a higher likelihood of seeking professional help for vignettes 1 and 2 (see table 2 and online supplemental table S2) in analyses controlling for age, gender, country of birth and level of education. Post hoc analyses of individual scale items are also given in table 2 and reveal that labelling was associated with intentions to speak to a mental health professional (GP, psychologist or psychiatrist) and to take medication. Labelling was associated with intentions to use phone counselling or an online mental health service only for vignette 1 (B=0.32 [99% CI 0.04, 0.61], p=0.003).

**Table 2:** Associations between depression labelling and behavioural intentions

	Currently well, family history	Currently well, own history	Subsyndromal depression	MDD	MDD with suicidal thoughts
	B (99% CI)	B (99% CI)	B (99% CI)	B (99% CI)	B (99% CI)
Professional help-seeking
Total	1.38 (0.46, 2.30)***	0.71 (0.02, 1.40)**	0.62 (−0.04, 1.28)	0.35 (−0.40, 1.08)	−0.02 (−0.81, 0.78)
Use telephone counselling/online support group	0.32 (0.04, 0.61)**	0.12 (−0.12, 0.37)	0.02 (−0.20, 0.24)	−0.04 (−0.29, 0.20)	−0.09 (−0.38, 0.19)
Use an online treatment programme	0.23 (0.05, 0.52)	0.06 (−0.17, 0.29)	0.01 (−0.21, 0.22)	−0.14 (−0.39, 0.10)	−0.21 (−0.47, 0.06)
Speak to a health professional	0.43 (0.16, 0.70)***	0.20 (−0.04, 0.43)	0.25 (0.04, 0.45)**	0.24 (0.10, 0.47)**	0.11 (−0.14, 0.37)
Take medication	0.40 (0.10, 0.70)**	0.33 (0.08, 0.58)**	0.34 (0.10, 0.57)***	0.30 (0.04, 0.55)**	0.19 (−0.09, 0.47)
Self help actions
Total	0.41 (−1.96, 2.78)	0.02 (−1.96, 2.00)	−0.77 (−2.63, 1.07)	−0.38 (−2.37, 1.60)	−2.06 (−4.56, 0.42)
Wait and see if you improve with time	0.02 (−0.22, 0.26)	−0.07 (−0.28, 0.15)	0.09 (−0.11, 0.29)	−0.01 (−0.22, 0.21)	−0.04 (−0.29, 0.22)
Try to avoid situations that make you feel anxious or uncomfortable	0.11 (−0.09, 0.31)	**0.14 (−0.03, 0.31)**	0.21 (0.05, 0.36)**	0.03 (−0.12, 0.18)	0.10 (−0.07, 0.28)

**p<0.01, ***p<0.001 (in bold type). Regression coefficients are unstandardised. All analyses control for age, gender, country of birth and level of education.

MDD, major depressive disorder.

There were no associations between depression labelling and total self-help total scores. Labelling depression was associated with an increased likelihood of trying to avoid situations that make you feel anxious or uncomfortable for vignette 2 (B=0.14 [99% CI −0.03, 0.31], p=0.034 and vignette 3 [ B=0.21 [99% CI 0.05, 0.36], p=0.001).

### Associations with psychological distress, and days out of role and self-reporting depression

**Figure 3:**
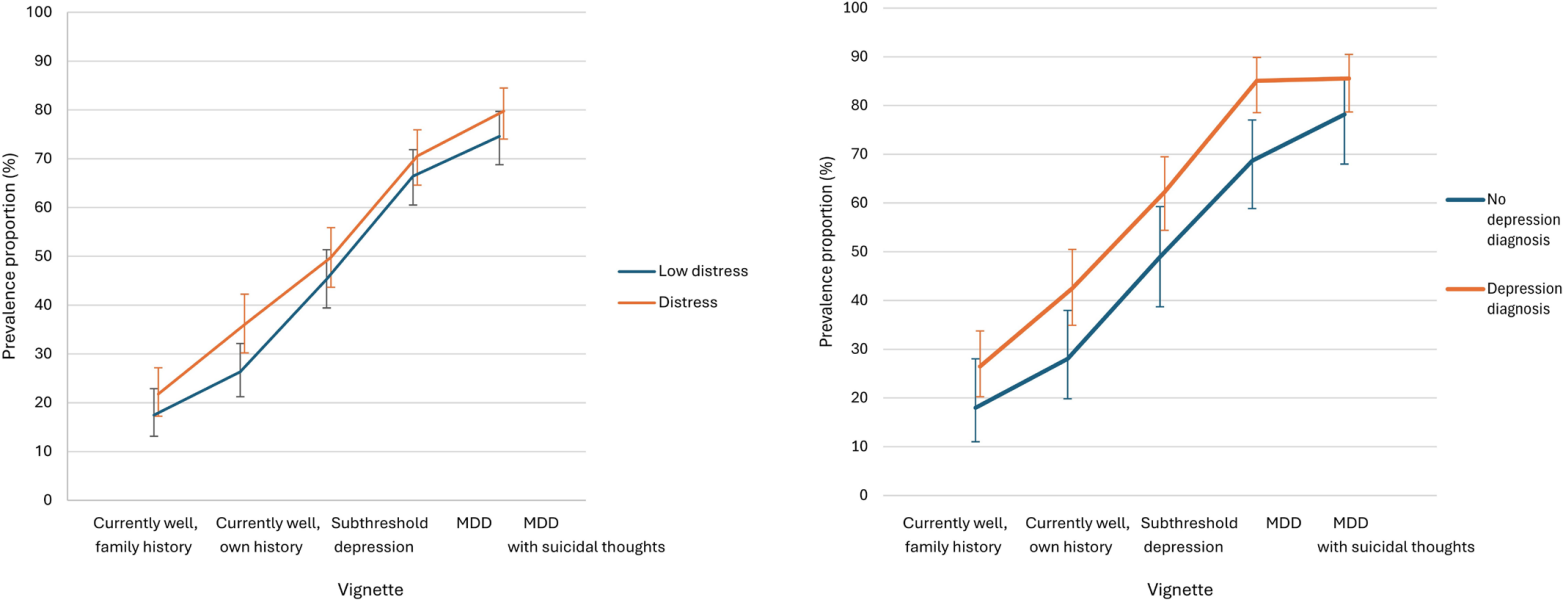
Associations between depression labelling and psychological distress and depression diagnosis. Error bars represent 99% CIs.

Labelling depression was not associated with a higher level of distress or self-reporting depression over the previous 12 months for any vignette (see figure 3). For vignette 2, labelling depression was associated with taking any days out of role (vs none) (OR=1.87 [99% CI 1.18, 2.95], p<0.001) and half days out of role (OR=2.00 [99% CI 1.31, 3.04], p<0.001) because of feelings of psychological distress.

## Discussion

We conducted the first large, rigorous, nationally representative survey exploring expanded concepts of depression and the associations between diagnostic labelling of non-clinical and subthreshold vignettes with psychological distress and intended actions. Labelling these vignettes with diagnostic labels was relatively common, with just under 20% of people labelling vignette 1 (currently well, family history) with a depression label and just under 50% labelling vignette 3 (subthreshold depressive symptoms). Labelling of non-clinical and subthreshold vignettes was most strongly associated with a greater likelihood of intending to speak to a health professional or take medication. Despite this, there was no support for associations between labelling and a reduction in intentions to take self-help actions. There were also no associations between labelling and psychological distress, although labelling vignette 2 was associated with days out of role because of those feelings. A minority did not correctly label vignettes 4 and 5, despite these vignettes clearly meeting the criteria for a diagnosis of depression.

### Associations with help-seeking and self-help beliefs

The associations between diagnostic labelling of non-clinical and subthreshold vignettes and professional help-seeking were largely driven by intentions to speak to a health professional and to take medication, even for vignettes 1 and 2, which describe a person as currently well. While this is in line with other recent studies,^25 38^ it does have implications for the mental health system, due to the risk of unnecessary treatment, including medication, or for resources being diverted from those with more severe illnesses.^24^ While participants may have preferred to ‘err on the side of caution’ and recommend speaking to a health professional even in the absence of symptoms, the intentions to use medication when labelling non-clinical vignettes 1 and 2 are particularly concerning and may partly reflect relatively high—and rising—levels of antidepressant prescribing in Australia.^39^ These findings point to the need for more nuance in the messaging directed towards people who may have mild symptoms, making it clear that some emotional and behavioural difficulties do not need to be labelled as mental illness or depression or be treated by a mental health professional or with medication.^14^ Given the key role of communication campaigns in efforts to raise awareness, it is perhaps not surprising that messaging has tended to be relatively simple, for example, “Mental illness is like any other medical illness”^40^ likening mental illness to a broken leg or encouraging people to visit their GP if they experience even transient psychological distress.^41^

A more nuanced approach will require a shift in how organisations promoting mental health literacy and help-seeking for mental health problems conduct their activities and, critically, careful testing to ensure that more nuanced messaging does not deter help-seeking from those who meet diagnostic criteria or widen help-seeking gaps in people from marginalised communities. In the current study, for example, only 68.6% [99% CI 64.5, 72.5] and 77.2% [99% CI 73.1, 80.7] labelled vignettes 4 and 5 with a depression label. These figures may represent decreases since the National Survey of Mental Health Literacy and Stigma in 2011.^31^ The finding in relation to vignette 5 is of particular concern given the suicide risk to the person in that vignette. Such messaging could, for example, strengthen the incorporation of education to raise awareness of evidence-based online early intervention and treatment programmes as, in the current study, diagnostic labelling was not associated with increased intentions to use these interventions. The Australian government funds many digital mental health services, which have been shown to be both effective and cost-effective,^42^ and there have been calls to develop information campaigns for the general public and health professionals to increase awareness and trust in these services.^43^ There have also been calls for non-medical early intervention and support services in the community, including those targeted to parents. Importantly, such services should not require diagnoses for access as this may further drive over-medicalisation.^14^ Campaigns could also incorporate further messaging on the importance of lifestyle interventions such as exercise, sleep hygiene and a diet high in unprocessed foods.^44^

### Sociodemographic characteristics and labelling

Younger people were generally more likely to label vignettes 2 and 3 with depression labels than those aged 65 and over, while there were fewer differences with the vignettes written to meet diagnostic criteria. This may be due to a general rise in cultural attention to depression and other harm concepts,^12^ accompanied by younger people having early and lifelong exposure to health education, as mental health education is now much more widespread in schools and in tertiary institutions.^45 46^ It is possible that early and continuous exposure to mental health education is linked to over-interpretation of symptoms.

Being born in a non-English-speaking country was associated with both lower labelling of vignette 3 and the vignettes written to meet diagnostic criteria. These findings are in line with previous studies showing lower levels of mental health literacy in people from culturally diverse backgrounds.^47^ They also point to the potential for increasing inequities in mental health service provision, as people from English-speaking backgrounds with milder problems may be more likely to access services than people born in non-English-speaking countries with more severe illness. Careful testing of campaign messaging promoting lower intensity or online services for milder problems is particularly critical to avoid further widening help-seeking gaps in people from non-English-speaking countries. Efforts to better engage and meet the needs of people from culturally diverse backgrounds are key to tackling this problem.

### Associations with psychological distress and days out of role

In this study, depression labelling was not associated with a higher level of distress for any vignette, suggesting that labelling does not drive distress. However, it is possible that labelling does increase sensitivity to previous episodes of poor mental health, as, for vignette 2, labelling was also associated with more days and half days out of role as well as greater intentions to ‘Look for patterns in when you feel anxious and how you react’ (B=0.17 [99% CI 0.1, 0.34], p=0.008) which may allude to ‘looping effects’^21^ and impacts on day-to-day activities. Further research using longitudinal designs is necessary to explore causality.

### Strengths and limitations

Strengths of the project include the fact that this is the first study to explore the issue of diagnostic expansion and its impacts using a large nationally representative sample. The key limitation is the cross-sectional nature of the survey, which precludes assessment of causality. A further limitation involves the use of vignettes, which naturally cannot fully represent complex human experiences related to experiencing a mental health problem. Moreover, vignettes 1, 2 and 3 contain less personal information about Sam than vignettes 4 and 5, potentially limiting comparability across vignettes that describe a person meeting diagnostic criteria and those that do not.

## Conclusions

While there are many benefits of increases in population mental health literacy, and increasing access to mental health services remains a pressing issue, findings from this study point to the need for public messages and narratives to have a more nuanced approach, making it clear that for some mental health difficulties, non-clinical solutions to distress may be more appropriate, while also taking care not to increase help-seeking gaps in those with more severe problems and in marginalised populations.

## Supplementary material

10.1136/bmjph-2025-003040 online supplemental file 1

10.1136/bmjph-2025-003040 online supplemental file 2

## Data Availability

Data are available upon reasonable request.
