# Supplementary material for: Testing the diagnostic expansion hypothesis with a population-based survey of attitudes to depression in Australia
Source: BMJ Public Health. 2025 Sep 9;3(2):e003040. doi: 10.1136/bmjph-2025-003040 (PMC12421594; doi:10.1136/bmjph-2025-003040)
Supplement: online supplemental file 1 [file bmjph-3-2-s001.docx]

## Appendix A: Vignettes

### Currently well, family history

Sam is 30 years old. He/she/they has/have recently been dealing with some stressful life events that are playing on his/her/their mind. This has affected his/her/their sleep a bit, although most nights he/she/they sleeps/sleep well. Because of these life events, he/she/they is/are finding it a bit difficult to keep his/her/their mind on his/her/their work. Sam also spends a lot of time with his/her/their mother who has a history of major depression.

### Currently well, own history

Sam is 30 years old. He/she/they has/have recently been dealing with some stressful life events that are playing on his/her/their mind. This has affected his/her/their sleep a bit, although most nights he/she/they sleeps/sleep well. Because of these life events, he/she/they is/are finding it a bit difficult to keep his/her/their mind on his/her/their work. Sam has had episodes of depression in the past.

### Subthreshold depressive symptoms

Sam is 30 years old. He/she/they has/have been feeling sad and miserable these past few weeks. He/she/they is/are not sleeping well. He/she/they is/are finding it difficult to keep his/her/their mind on his/her/their work. This has come to the attention of his/her/their boss, who is concerned about his/her/their lowered productivity.

### Meets criteria for MDD

Sam is 30 years old. He/she/they has/have been feeling unusually sad and miserable for the last few weeks. Even though he/she/they is/are tired all the time, he/she/they has/have trouble sleeping nearly every night. Sam doesn't feel like eating and has lost weight. He/she/they can't keep his/her/their mind on his/her/their work and puts off making decisions. Even day-to-day tasks seem too much for him/her/them. This has come to the attention of his/her/their boss, who is concerned about Sam’s lowered productivity.

### MDD with suicidal thoughts

Sam is 30 years old. He/she/they has/have been feeling unusually sad and miserable for the last few weeks. Even though he/she/they is/are tired all the time, he/she/they has/have trouble sleeping nearly every night. Sam doesn't feel like eating and has lost weight. He/she/they can't keep his/her/their mind on his/her/their work and puts off making decisions. Even day-to-day tasks seem too much for him/her/them. This has come to the attention of his/her/their boss, who is concerned about Sam’s lowered productivity. Sam feels he/she/they will never be happy again and believes his/her/their family would be better off without him/her/them. Sam has been so desperate, he/she/they has been thinking of ways to end his/her/their life.
