# Supplementary material for: Testing the diagnostic expansion hypothesis with a population-based survey of attitudes to depression in Australia
Source: BMJ Public Health. 2025 Sep 9;3(2):e003040. doi: 10.1136/bmjph-2025-003040 (PMC12421594; doi:10.1136/bmjph-2025-003040)
Supplement: online supplemental file 2 [file bmjph-3-2-s002.docx]

## Supplementary Tables

*Table 1 Sociodemographic characteristics associated with depression labelling*

|  | Currently well, family history |  | Currently well, own history |  | Subsyndromal depression |  | MDD |  | MDD with suicidal thoughts |  |
| --- | --- | --- | --- | --- | --- | --- | --- | --- | --- | --- |
|  | Odds Ratio (99%CI) | p | Odds Ratio (99%CI) | p | Odds Ratio (99%CI) | p | Odds Ratio (99%CI) | p | Odds Ratio (99%CI) | p |
| **Gender (reference category: male)** | | | | | | | | | | |
| **Female** | 1.14 [0.73,1.79] | 0.445 | 1.20 [0.81,1.78] | 0.226 | 1.36 [0.95,1.95] | 0.028 | **1.59 [1.06,2.36]** | **0.003** | **1.64 [1.04,2.59]** | **0.005** |
| **Prefer another term** | 1.33 [0.08,23.22] | 0.799 | 2.68 [0.39,18.29] | 0.185 | 3.48 [0.46,26.48] | 0.113 | 1.67 [0.33,8.35] | 0.412 | 1.00 [0.00,0.00] | - |
|  | | | | | | | | | | |
| **Age groups (reference category: age 65 plus)** | | | | | | | | | | |
| 16-34 | 1.54 [0.78,3.02] | 0.101 | **1.77 [1.01,3.10]** | **0.009** | **1.85 [1.10,3.12]** | **0.002** | **2.19 [1.26,3.81]** | **<0.001** | 1.32 [0.71,2.45] | 0.253 |
| 35-64 | 1.33 [0.74,2.40] | 0.215 | 0.99 [0.61,1.59] | 0.952 | 1.40 [0.92,2.13] | 0.038 | 1.49 [0.94,2.36] | 0.025 | **1.71 [1.02,2.88]** | **0.007** |
|  | | | | | | | | | | |
| **Country of birth (reference category: Australian born)** | | | | | | | | | | |
| Non-English-speaking countries | 0.87 [0.46,1.63] | 0.559 | 1.00 [0.59,1.70] | 0.992 | **0.32 [0.20,0.53]** | **<0.001** | **0.52 [0.31,0.87]** | **0.001** | **0.36 [0.21,0.60]** | **<0.001** |
| Main English-speaking countries | 1.07 [0.53,2.14] | 0.810 | 1.27 [0.71,2.27] | 0.284 | 0.81 [0.48,1.37] | 0.297 | 0.92 [0.51,1.67] | 0.72 | 1.27 [0.60,2.68] | 0.415 |
|  |  |  |  |  |  |  |  |  |  |  |
| **Mental health training (reference category: no training)** | | | | | | | | | | |
| Health professional training | 0.95 [0.49,1.85] | 0.84 | 0.97 [0.53,1.76] | 0.896 | 1.50 [0.80,2.81] | 0.095 | 2.01 [0.91,4.44] | 0.023 | 1.41 [0.65,3.07] | 0.253 |
| Mental health/ suicide prevention training | 1.05 [0.53,2.06] | 0.866 | 1.50 [0.86,2.63] | 0.059 | 1.49 [0.87,2.52] | 0.054 | 1.21 [0.65,2.24] | 0.435 | 0.91 [0.42,2.01] | 0.77 |

*Table 2 Associations between depression labelling and behavioural intentions*

|  | Currently well, family history |  | Currently well, own history |  | Subsyndromal depression |  | MDD |  | MDD with suicidal thoughts |  |
| --- | --- | --- | --- | --- | --- | --- | --- | --- | --- | --- |
|  | B (99%CI) | p | B (99%CI) | p | B (99%CI) | p | B (99%CI) | p | B (99%CI) | p |
|  | | | | | | | | | | |
| **Professional help-seeking total** | **1.38 [0.46,2.30]** | **<0.001** | **0.71 [0.02,1.40]** | **0.008** | 0.62 [-0.04,1.28] | 0.016 | 0.35 [-0.40,1.08] | 0.230 | -0.02 [-0.81,0.78] | 0.956 |
| *- Use telephone counselling/ online support group* | **0.32 [0.04,0.61]** | **0.003** | 0.12 [-0.12,0.37] | 0.198 | 0.02 [-0.20,0.24] | 0.789 | -0.04 [-0.29,0.20] | 0.643 | -0.09 [-0.38,0.19] | 0.386 |
| *- Use an online treatment program* | 0.23 [0.05,0.52] | 0.037 | 0.06 [-0.17,0.29] | 0.478 | 0.01 [-0.21,0.22] | 0.957 | -0.14 [-0.39,0.10] | 0.140 | -0.21 [-0.47,0.06] | 0.045 |
| *- Speak to a health professional* | **0.43 [0.16,0.70]** | **<0.001** | 0.20 [-0.04,0.43] | 0.030 | **0.25 [0.04,0.45]** | **0.003** | **0.24 [0.10,0.47]** | **0.008** | 0.11 [-0.14,0.37] | 0.257 |
| *- Take medication* | **0.40 [0.10,0.70]** | **0.001** | **0.33 [0.08,0.58]** | **0.001** | **0.34 [0.10,0.57]** | **<0.001** | **0.30 [0.04,0.55]** | **0.003** | 0.19 [-0.09,0.47] | 0.082 |
|  |  |  |  |  |  |  |  |  |  |  |
| **Self-help actions total** | 0.41 [-1.96,2.78] | 0.657 | 0.02 [-1.96,2.00] | 0.981 | -0.77 [-2.63,1.07] | 0.281 | -0.38 [-2.37,1.60] | 0.616 | -2.06 [-4.56,0.42] | 0.032 |
|  |  |  |  |  |  |  |  |  |  |  |
| Wait and see if you improve with time | 0.02 [-0.22.0.26] | 0.829 | -0.07 [-0.28,0.15] | 0.426 | 0.09 [-0.11,0.29] | 0.261 | -0.01 [-0.22,0.21] | 0.929 | -0.04 [-0.29,0.22] | 0.703 |
| Try to avoid situations that make you feel anxious or uncomfortable | 0.11 [-0.09,0.31] | 0.142 | **0.14 [-0.03.0.31]** | **0.034** | **0.21 [0.05.0.36]** | **0.001** | 0.03 [-0.12,0.18] | 0.645 | 0.10 [-0.07,0.28] | 0.130 |

Note: Regression coefficients are unstandardised. All analyses control for age, gender, country of birth and level of education
